# Nipah Virus Infection of Immature Dendritic Cells Increases Its Transendothelial Migration Across Human Brain Microvascular Endothelial Cells

**DOI:** 10.3389/fmicb.2018.02747

**Published:** 2018-11-13

**Authors:** Vunjia Tiong, Meng-Hooi Shu, Won Fen Wong, Sazaly AbuBakar, Li-Yen Chang

**Affiliations:** ^1^Department of Medical Microbiology, Faculty of Medicine, University of Malaya, Kuala Lumpur, Malaysia; ^2^Tropical Infectious Diseases Research and Education Centre, University of Malaya, Kuala Lumpur, Malaysia

**Keywords:** nipah virus (NiV), transendothelial migration, *in vitro* blood brain barrier, immature dendritic cells, monocytes, Trojan horse

## Abstract

Nipah virus (NiV) can infect multiple organs in humans with the central nervous system (CNS) being the most severely affected. Currently, it is not fully understood how NiV spreads throughout the body. NiV has been shown to infect certain leukocyte populations and we hypothesized that these infected cells could cross the blood-brain barrier (BBB), facilitating NiV entry into the CNS. Here, three leukocyte types, primary immature dendritic cells (iDC), primary monocytes (pMO), and monocytic cell line (THP-1), were evaluated for permissiveness to NiV. We found only iDC and THP-1 were permissive to NiV. Transendothelial migration of mock-infected and NiV-infected leukocytes was then evaluated using an *in vitro* BBB model established with human brain microvascular endothelial cells (HBMEC). There was approximately a threefold increase in migration of NiV-infected iDC across endothelial monolayer when compared to mock-infected iDC. In contrast, migration rates for pMO and THP-1 did not change upon NiV infection. Across TNF-α-treated endothelial monolayer, there was significant increase of almost twofold in migration of NiV-infected iDC and THP-1 over mock-infected cells. Immunofluorescence analysis showed the migrated NiV-infected leukocytes retained their ability to infect other cells. This study demonstrates for the first time that active NiV infection of iDC and THP-1 increased their transendothelial migration activity across HBMEC and activation of HBMEC by TNF-α further promoted migration. The findings suggest that NiV infection of leukocytes to disseminate the virus via the “Trojan horse” mechanism is a viable route of entry into the CNS.

## Introduction

Nipah virus (NiV), an emergent fatal paramyxovirus of the genus Henipavirus, first emerged in late 1998 in Malaysia and caused an outbreak of acute encephalitis resulting in 265 reported cases and 105 deaths (Chua et al., 2000). In addition to a broad host range, NiV is able to infect multiple organs such as heart, kidney, lungs, and brain. This is due to the viral entry receptors ephrinB2 and ephrinB3 being expressed on multiple cell types, and which are also highly conserved across mammalian species (Wong et al., 2002; Bonaparte et al., 2005; Negrete et al., 2005, 2006; Wang et al., 2013). In humans, the central nervous system (CNS) was the most severely affected organ in patients in the Malaysia outbreak (Wong et al., 2002), while severe respiratory disease was the primary manifestation in the Bangladesh outbreaks (Chadha et al., 2006; Chong et al., 2008; Sazzad et al., 2013). It is currently not fully understood how NiV efficiently spreads to different organs in an infected host, especially to the CNS.

The presence of the blood-brain barrier (BBB) tightly regulates movement of solutes, molecules and cells, and aims to restrict entry of pathogens into the brain (Abbott et al., 2010; Spindler and Hsu, 2012). However, the BBB can be breached by direct virus infection of the cerebral blood vessels, by actions of viral proteins which degrade BBB components, and by migration of infected leukocytes across the BBB by a “Trojan horse” mechanism (Verma et al., 2009; Strazza et al., 2011). An alternative pathway for viral entry besides crossing the BBB is by axonal spread of virus into the CNS (Gillet et al., 1986; Chen et al., 2007; Samuel et al., 2007; McGraw et al., 2009). For NiV, studies on its spread in an infected host are limited and current understanding stems from infection in animal models. In hamsters, NiV was found to initially target the respiratory system, specifically the olfactory epithelium (Baseler et al., 2016), and was shown to utilize the olfactory route traveling along neurons in the olfactory epithelium into the brain (Munster et al., 2012). In the porcine model, NiV was found to spread via the cranial nerves and hematogenously to the CNS (Weingartl et al., 2005). Alternatively, NiV may utilize leukocytes for virus spread. A previous study showed low level NiV replication in a monocytic cell line (THP-1) (Chang et al., 2006), and more recently the same was demonstrated in immature dendritic cells (Mathieu et al., 2011). It was reported that lymphocytes and monocytes were able to bind NiV on their cell surface and cause *trans*-infection *in vitro* and in hamsters (Mathieu et al., 2011). NiV may also utilize the “Trojan horse” mechanism where NiV-infected leukocytes exit the blood vessels to cause infection in the surrounding tissues. This process of movement across the endothelium of blood vessels is termed diapedesis or transendothelial migration, and is highly regulated as recruited leukocytes undergo a cascade of steps to cross the endothelium, basement membrane and into the interstitial tissues (Muller, 2009). Infection of leukocytes by certain viruses has shown to alter its transendothelial migration activity and facilitate viral dissemination. For example, infection of lymphocytes by measles virus, also a paramyxovirus, increased expression of surface adhesion molecules, resulting in a stronger binding of the lymphocytes to endothelial cells and inhibiting lymphocyte migration thus enabling transmission of the virus from the lymphocytes to the endothelial cells (Dittmar et al., 2008). On the other hand, HIV infection of leukocytes enhanced their response to chemokine CCL2, increasing their migration across an *in vitro* human BBB model for CNS invasion (Eugenin et al., 2006). To date, the migration activity of NiV-infected leukocytes has not yet been reported.

We hypothesized that NiV-infected leukocytes could use the “Trojan horse” mechanism of virus spread to enter the CNS. For this, we evaluated human monocytes and immature dendritic cells for their permissiveness to NiV. These cells are present in the blood and, if able to support NiV infection, would likely aid virus spread throughout the body. The NiV-infected leukocytes were then evaluated for changes in their transendothelial migration activity across an *in vitro* BBB model. The *in vitro* BBB model was established using primary human brain endothelial cells. NiV infection was found to markedly increase the transendothelial migration of primary immature dendritic cells (iDC), which was permissive to NiV replication. TNF-α treatment of the endothelial cells was found to enhance migration of NiV-infected leukocytes. Besides, the migrated NiV-infected leukocytes retained their infectivity. We report here for the first time that NiV infection of leukocytes increased their transendothelial migration activity, which may facilitate virus dissemination in a host.

## Materials and Methods

### Cells and Virus

Cell lines THP-1 (ATCC No. TIB-202) and Vero (ATCC No. CCL-81) were purchased from the American Type Culture Collection, United States and maintained in RPMI 1640 (HyClone, United States) and EMEM (Gibco, United States), respectively, supplemented with 10% FBS (Bovogen, Australia), 2 mM L-glutamine (Gibco, United States) and 1 × non-essential amino acid (Gibco, United States). For THP-1, the media was also supplemented with 0.05 mM 2-mercaptoethanol (Gibco, United States). The cells were maintained at 37°C in a humidified incubator containing 5% CO_2_. Primary human brain microvascular endothelial cells (HBMEC), human umbilical vein endothelial cells (HUVEC) and the endothelial cell complete media were purchased from ScienCell Research Laboratories, United States. Human astrocytes and its complete media were also purchased from ScienCell Research Laboratories, United States.

The NiV strain NV/MY/99/VRI-2794 was obtained from the Department of Medical Microbiology, Faculty of Medicine, University of Malaya. This strain was isolated from pigs during the 1998 outbreak in Malaysia (Genbank: AJ564621) (AbuBakar et al., 2004). The virus, obtained at passage 4, was further propagated in Vero cells to yield a virus stock which was used in this study. Briefly, NiV-infected Vero cells were harvested upon 80% cytopathic effect (CPE), i.e., syncytial formation, and centrifuged at 800 ×*g*. The clarified supernatant was aliquoted, labeled and stored securely at -80^o^C. This NiV stock was passage 5 and was titrated by plaque assay. Briefly, 10-fold serial dilutions of NiV stock were prepared and added into 24-well culture plate seeded with Vero monolayer, and incubated at 37^o^C, 5% CO_2_ for 3.5 days. The cells were then fixed with 4% paraformaldehyde for 1 h at room temperature and overnight at 4^o^C, and stained with 1% (w/v) crystal violet prepared in 20% ethanol for 30 min at room temperature. The stain was discarded and wells were washed twice with phosphate-buffered saline (PBS), followed by a final wash with distilled water. The number of plaques formed was counted using the Nikon SMZ1000 stereomicroscope (Nikon, Japan), and the virus titer in plaque forming units per ml (pfu/ml) was determined based on two independently performed plaque assays.

All experiments that involved handling of live NiV were conducted at the Biosafety Level 3 (BSL3) facility at the Tropical Infectious Diseases Research and Education Centre (TIDREC), University of Malaya. NiV is classified as a risk group 3 agent in Malaysia (Prevention and Control of Infectious Diseases Act 1988, Malaysia) and biorisk assessment was performed to determine the control measures required to minimize laboratory-acquired infection.

### Isolation of PMBC

Human peripheral blood mononuclear cells (PBMC) were isolated by density gradient centrifugation using Ficoll-Paque PLUS (GE Healthcare, United States) from whole blood collected and pooled from four healthy donors with no known history of NiV exposure. The protocol was approved by the University of Malaya Medical Centre Ethics Committee, MECID No. 20164-2360. PBMC obtained from the interface were washed three times in Dulbecco’s PBS (Gibco, United States). Primary monocytes (pMO) were purified from the PBMC by negative selection using the Pan Monocyte Isolation Kit from MACS Miltenyi Biotec, United States. To obtain immature dendritic cells (iDC), pMO were stimulated with 40 ng/ml GM-CSF (Gibco, United States) and 40 ng/ml IL-4 (Gibco, United States). The media was replaced on the 3rd and 5th day with media containing the same concentration of GM-CSF and IL-4. On the 8th day after differentiation, iDC were collected and used in subsequent analyses. Both pMO and iDC were maintained in RPMI 1640 (HyClone, United States), 10% FBS, 2 mM L-glutamine and 1× non-essential amino acid. To ascertain differentiation to iDC, the cells on Day 8 were harvested by centrifugation at 200 ×*g* and incubated with antibodies against CD14-FITC, CD1a-PE and CD83-APC (BD Biosciences, United States) for 30 min at room temperature in the dark. Cells were then washed twice by centrifugation with FACS buffer (2% FBS, 1% 0.5 M EDTA, 0.1% sodium azide in PBS), resuspended in 150 μl FACS buffer, and analyzed using the FACSCANTO^TM^ II (BD Biosciences, United States).

### NiV Replication Kinetics

To examine the NiV replication kinetics in iDC, pMO, and THP-1, these cells were infected with NiV at a multiplicity of infection (MOI) 2 at the same cell number. Briefly, virus inoculum was added to the cells and the virus was allowed to pre-absorb to the cells for 1 h at room temperature. The cells were then washed twice with serum-free RPMI 1640 by centrifugation at 200 ×*g* to remove the inoculum and resuspended in fresh RPMI 1640 containing 2% FBS. At pre-determined time points post-infection (p.i.), every 24 h until 96 h p.i., the cells were harvested and washed twice by centrifugation. TRI reagent^®^ or TRI reagent LS^®^ (Molecular Research Center Inc., United States) was added to the cell pellet (to assess intracellular viral RNA level) and cell supernatant (to assess extracellular viral RNA level), respectively. Total RNA was then extracted following the manufacturer’s protocol. The RNA pellet was dissolved in nuclease-free water. To remove any genomic DNA leftover, the RNA was treated with RQ1 RNase-free DNase (Promega, United States) at 37^o^C for 30 min and RQ1 stop solution (Promega, United States) was added thereafter, following manufacturer’s protocol. To determine the NiV N gene copy number, TaqMan^®^ Fast Virus 1-Step Master Mix and Custom TaqMan^®^ Gene Expression assay were used (Applied Biosystems, United States). The 12 μl reaction mix consisted of 4× TaqMan^®^ Fast Virus 1-Step Master Mix (3 μl), 20× custom TaqMan^®^ Gene Expression assay (0.6 μl), RNA template (3 μl) and nuclease-free water (5.4 μl). The following cycling conditions were performed using the StepOnePlus^TM^ instrument (Applied Biosystems, United States): reverse transcription at 50^o^C for 5 min; RT inactivation at 95^o^C for 20 s; 40 cycles of amplification at 95^o^C for 3 s and 60^o^C for 30 s. Details of the primers are as follows: forward primer: 5′ ATCGGAAACTATGTCGAGGAAACTG 3′, reverse primer: 5′ CTCCAACCCGAATCTGATGGT 3′, reporter: 5′ ATGGCAGGATTCTTCG 3′. For the standard, a segment of the NiV nucleocapsid (N) gene encompassing the amplified region was *in vitro* transcribed and purified by lithium chloride precipitation following manufacturer’s protocol (Invitrogen, United States). The integrity of the transcribed, purified RNA was verified and quantified using the 2100 Bioanalyzer (Agilent, United States), and the NiV N copy number/ml was calculated from the average of two independently performed replicates.

### Immunofluorescence Assay

To visualize and confirm infection of iDC, pMO, and THP-1 by NiV, the cells were analyzed by immunofluorescence staining. The cells infected with NiV were fixed in 4% paraformaldehyde for 1 h at room temperature and then overnight at 4^o^C. The fixed cells were added to Poly-L-lysine-coated glass slides, permeabilized with 0.1% Triton X-100/PBS for 10 min and blocked with 3% bovine serum albumin (Sigma-Aldrich, United States) for 30 min. The cells were incubated with mouse polyclonal IgG antibody against NiV N protein (Tiong et al., 2017) at 1:50 dilution for 1 h at room temperature, washed three times with PBS and followed by incubation with a secondary antibody of either Alexa Fluor 488 or Alexa Fluor 546 (Invitrogen, United States) at 1:1000 dilution for 1 h at room temperature. Cell nuclei were counterstained with Hoechst 33342 (Invitrogen, United States). Slides were mounted with ProLong^®^ Gold anti-fade reagent (Invitrogen, United States) and viewed under the Nikon TE200 inverted fluorescence microscope (Nikon, Japan). Analysis was performed from at least three high-powered fields (200× magnification).

### Endogenous mRNA Expression of EphrinB2 and EphrinB3

To assess the mRNA expression of ephrinB2 and ephrinB3 in cells, total RNA was extracted from iDC, pMO, THP-1, and Vero as described earlier. Similarly, any leftover genomic DNA was removed by treatment with RQ1 RNase-free DNase. The total RNA was quantified using a nanospectrophotometer (Implen, Germany). Generation of cDNA was performed using SuperScript^®^ III Reverse Transcriptase (Invitrogen, United States). The reaction mix consisted of 50 ng RNA template, 50 μM oligo(dT)18 mRNA primer (1 μl), 10 mM dNTP mix (1 μl) and nuclease-free water (to a final volume of 13 μl). This mixture was incubated at 65°C for 5 min and then placed on ice for at least 1 min. To this, 5× RT buffer (4 μl), 0.1 M DTT (1 μl), 200 units/μl SuperScript^TM^ III RT (1 μl) and nuclease-free water (1 μl) were added, yielding a final reaction volume of 20 μl. The cycling conditions were 25°C for 5 min, 50°C for 60 min, and 70°C for 15 min on the Veriti^®^ Thermal Cycler (Applied Biosystems, United States). The cDNA was then used for PCR to amplify ephrinB2, ephrinB3, and β-actin mRNA. The PCR reaction mix consisted of 60 ng cDNA template (3 μl), 10× Dream Taq buffer (1.25 μl), 25 mM MgCl_2_ (0.5 μl), 10 mM dNTP mix (0.25 μl), 10 pM forward primer (0.25 μl), 10 pM reverse primer (0.25 μl), DreamTaq DNA polymerase (Thermo Fisher Scientific, Waltham, Massachusetts, United States) (0.25 μl) and nuclease-free water (6.75 μl), yielding a final reaction volume of 12.5 μl. Primers used were: ephrinB2 forward 5′ GAAAATACCCCTCTCCTCAACT 3′, reverse 5′ CTTCGGAACCGAGGATGTTGTTC 3′ (394 bp) (Williams et al., 2006); ephrinB3 forward 5′ CCAGGCAGAGGGTGGTTATG 3′, reverse 5′ TCAGACACAGGTTTTCGGGG 3′ (423 bp) (this work); β-actin forward 5′ GGGTCAGAAGGATTCCTATG 3′, reverse 5′ GGTCTCAAACATGATCTGGG 3′ (237 bp) (Marceau et al., 2004). An annealing temperature of 50°C was used for ephrinB2 and β-actin primers while 55°C was used for ephrinB3 primers. The cycling conditions were: 94°C for 2 min; 40 cycles of 94°C for 30 s, 50/55°C for 30 s, 72°C for 1 min; and final extension at 72°C for 5 min. All PCR reactions were performed using the Veriti^®^ Thermal Cycler. The amplified DNA fragments were separated by electrophoresis on a 1.8% (w/v) agarose gel. For each sample, two independently performed amplifications were carried out.

### *In vitro* Blood-Brain Barrier (BBB) Model

Astrocyte-conditioned medium (ACM) was prepared by growing human astrocytes, from passages 4 or 5, in a 75 cm^2^ culture flask with 15 ml complete media until 90–100% confluent. Then, the spent media (15 ml) was discarded and replaced with fresh astrocyte media. After 24 h, the entire culture supernatant was harvested, centrifuged to remove cell debris and passed through a 0.45 μm filter, aliquoted and stored as ACM at -80^o^C until further use. ACM was used as it has been shown to help maintain BBB characteristics *in vitro* (Abbott, 2002; Patabendige et al., 2013; Helms et al., 2016).

An *in vitro* BBB model was established using a 5 μm pore size transwell (Corning, United States). The transwells were first coated for 2 h each with 45 μg/cm^2^ Type 1 rat tail collagen (Sigma-Aldrich, United States) followed by 4 μg/cm^2^ fibronectin (Sigma-Aldrich, United States). HBMEC were then seeded at 3.2 × 10^4^ cells/transwell and incubated for 3 days at 37^o^C in a humidified chamber containing 5% CO_2_. The media in the upper and lower chamber were ECM with 5% FBS and ACM at a ratio 1:1 supplemented with 100 μg/ml heparin (Sigma-Aldrich, United States) and 550 nM hydrocortisone (Sigma-Aldrich, United States). On the 4th day, media in the upper and lower chamber was replaced with serum-free ECM, 100 μg/ml heparin, 550 nM hydrocortisone, 250 μM cAMP (Sigma-Aldrich, United States), and 17.5 μM RO-20-1724 (Calbiochem, United States), and further incubated for 24 h. The *in vitro* BBB models were used for transendothelial migration assay the next day. The transendothelial electrical resistance (TEER) of the endothelial cell monolayer was measured every day using the EVOM2 voltohmmeter from World Precision Instruments, United States. HUVEC were seeded in the same manner for comparison in the transendothelial migration assay.

### Transendothelial Migration Assay

For the transendothelial migration assay, mock-infected and NiV-infected leukocytes were allowed to migrate across endothelial cell monolayers, specifically HBMEC, HUVEC, TNF-α-treated HBMEC (HBMEC/TNF-α) and TNF-α-treated HUVEC (HUVEC/TNF-α). iDC, pMO and THP-1 were infected with NiV at MOI 2 as described earlier. The leukocytes were infected for 48 h prior to the migration assay. The infection period of 48 h was arbitrarily chosen to ensure virus replication was underway in these cells. At 48 h p.i., the leukocytes were washed twice with serum-free RPMI 1640 to remove any extracellular viruses and then resuspended in migration media (serum-free ECM:RPMI 1640 at 1:1 ratio). To examine migration across TNF-α-treated endothelial cells, the endothelial cells were first treated for 4 h with TNF-α (Gibco, United States) at 2 ng/ml prepared in serum-free ECM. The media containing TNF-α was then removed and transwells were washed twice with Hanks’ balanced salt solution. Thereafter, media in the lower chamber was replaced with 950 μl migration media supplemented with 50 ng/ml RANTES (Gibco, United States) and 50 ng/ml MCP-1 (Gibco, United States) as chemoattractants. In the upper chamber, 150 μl of approximately 2 × 10^5^ mock-infected or NiV-infected leukocytes in migration media only was added. The plates with the transwells were incubated at 37^o^C for 6 h to allow cell migration to occur. To harvest migrated leukocytes, the bottom of the transwell was gently rinsed with serum-free ECM to release any leukocytes that had migrated across the endothelial monolayer but had not yet dropped to the lower chamber. A cell scrapper was used to gently remove any leukocytes which may have adhered to the well surface in the lower chamber. A total of 900 μl of media containing migrated leukocytes from the lower chamber was then collected and 600 μl of 10% paraformaldehyde was added to obtain a final paraformaldehyde concentration of 4% for sample fixation overnight (1 h at room temperature followed by overnight at 4^o^C). The transwells were then immediately transferred to a new 24-well plate to perform the permeability assay. The migration assay was performed with three biological replicates and in at least two independent experiments.

To count the migrated leukocytes, samples from the lower chamber which were previously fixed in paraformaldehyde overnight were centrifuged at 500 ×*g* for 10 min. The supernatant was discarded to yield a remaining volume of 100 μl. From there, 10 μl of the leukocyte suspension was removed for counting using a hemocytometer. Each sample was counted at least five times and the average taken. This represented the number of migrated leukocytes across the *in vitro* BBB model. Migration was presented as the percentage of total cells added to the transwell: Migration (%) = (number of migrated cells)/(total number of cells added) × 100%.

### Permeability Assay

To evaluate the permeability of endothelial cells in the transwell immediately after migration, transwells from the migration assay were transferred to a new 24-well plate containing 950 μl phenol red-free, serum-free ECM in the lower chamber. To the upper chamber, 150 μl of 0.25 M fluorescein-dextran (Invitrogen, United States), prepared in the same medium, was added. The plate was returned to 37°C. After 1 h, the transwells were carefully removed from each well, and 100 μl of media from the lower chamber was removed. The media was transferred to a round-bottom 96-well plate to determine the flux of fluorescein-dextran across the endothelial monolayer as measured using a fluorescence plate reader (Tecan, United States). Each well was sampled three times and the average fluorescence reading was taken. The permeability assay, as a continuation from the migration assay, was performed with three biological replicates and in at least two independent experiments.

### Overlay of Migrated Cells on Vero Cells

To evaluate if the migrated leukocytes infected with NiV could still infect other susceptible cells, the leukocytes collected after transendothelial migration assay were overlaid on a Vero cell monolayer. Falcon culture slides (BD Falcon, United States) were seeded with 1.6 × 10^5^ Vero cells the day before to achieve a monolayer the following day. After transendothelial migration, the migrated leukocytes were harvested and pooled from the lower chamber of three transwells. The supernatant was removed by centrifugation and the leukocyte cell pellet was resuspended in 200 μl serum-free RPMI containing 0.5 μM CellTracker^TM^ Green CMFDA (Invitrogen, United States) for 45 min. CMFDA (5-chloromethylfluorescein diacetate) dye, when added to the leukocytes, would pass through the cell membrane into the cell and is converted to fluorescent cell membrane-impermeant products. This was used to differentiate the leukocytes from the unstained Vero cell monolayer. The leukocytes were then washed twice to remove the extracellular fluorescent dye and resuspended in serum-free RPMI. The leukocytes were added to the Vero cell monolayer in the Falcon culture slides and incubated at 37°C for 24 h, after which the cells were fixed with 4% paraformaldehyde overnight at 4°C. The leukocytes and Vero cells were then subjected to immunofluorescence staining as described earlier. This analysis was performed for all replicates of the migration assay mentioned above.

### Statistics

GraphPad Prism 5 software (GraphPad Software Inc., United States) was used to perform all statistical analysis and graphical representations. Data analysis was performed using one-way analysis of variance (ANOVA) with Bonferroni method for comparison of three or more groups.

## Results

### NiV Replication Kinetics in Leukocytes

**FIGURE 1:**
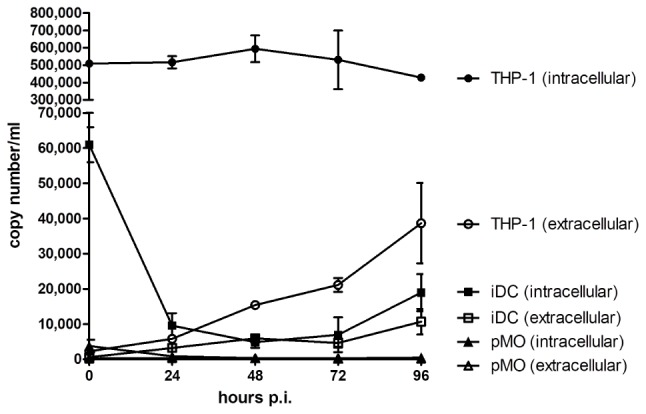
Evaluation of NiV replication kinetics in iDC, pMO, and THP-1 infected with NiV. The extracellular and intracellular NiV N copy numbers were determined by real-time qPCR from 0 to 96 h p.i. Values are the average of two technical replicates and error bars indicate one standard deviation. Data is representative of two biological replicates.

Three different human leukocytes were evaluated for their permissibility to NiV infection: iDC, pMO, and THP-1. The replication kinetics of NiV in infected leukocytes were determined by qPCR (Figure 1). iDC and THP-1 were found to support low level NiV replication. In iDC, the extracellular NiV N gene copy number increased to around 1 × 10^4^ copy number/ml at 96 h p.i., while in THP-1, the extracellular copy number increased to around 4 × 10^4^ copy number/ml after the same period. The intracellular NiV N copy number in iDC decreased after 0 h (time counted after pre-adsorption) from approximately 6 × 10^4^ copy number/ml to 1 × 10^4^ copy number/ml at 48 h p.i., and increased to nearly 2 × 10^4^ copy number/ml at 96 h p.i. In THP-1, the intracellular NiV N copy number stayed level at approximately 5 × 10^5^ copy number/ml from 0 to 24 h p.i., peaked at nearly 6 × 10^5^ copy number/ml at 48 h p.i., and decreased thereafter. The intracellular NiV N copy number in THP-1 was approximately one log higher than in iDC. In contrast, a decreasing trend in extracellular and intracellular NiV N copy number was observed in pMO up until 96 h p.i.

### Endogenous NiV Entry Receptor Expression in Leukocytes

**FIGURE 2:**
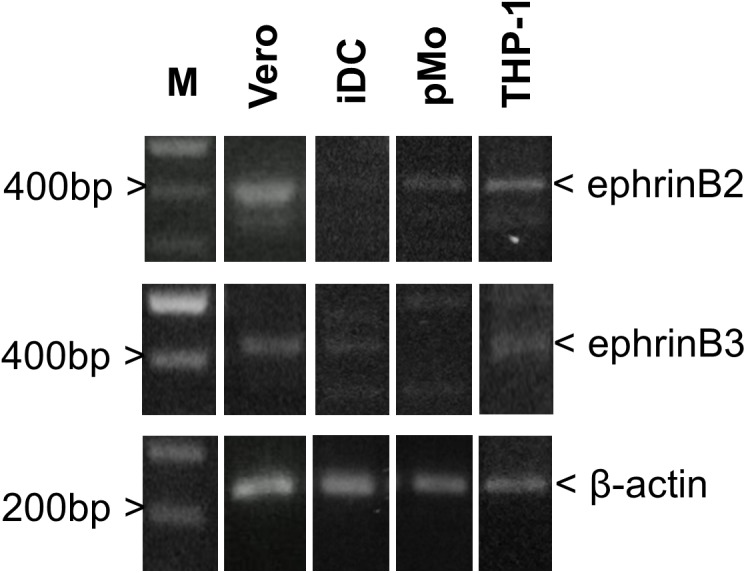
PCR amplification for detection of endogenous mRNA levels of ephrinB2, ephrinB3 and β-actin in iDC, pMO, THP-1, and Vero cells. Amplified products were visualized on a 1.8% (w/v) agarose gel. Lane M – GeneRuler 100 bp DNA Ladder Plus (Promega, United States). Figure shows the representative results from two independently performed amplifications.

Endogenous mRNA levels of ephrinB2 and ephrinB3 in iDC, pMO, and THP-1 were compared (Figure 2) and intensity of the bands were analyzed using ImageJ software (Schneider et al., 2012; Supplementary Material). The endogenous mRNA levels of the housekeeping gene β-actin served as the control. Vero cells, which are susceptible and permissive to NiV, were used as a comparison. EphrinB2 mRNA was detected in iDC, pMO, and THP-1 although the band intensity was faint for iDC. EphrinB3 mRNA was detected faintly in iDC and THP-1, but almost absent in pMO. Both ephrinB2 and ephrinB3 mRNA were noted in Vero cells. Overall, mRNA levels of ephrinB2 and ephrinB3 in the human leukocytes were much lower than that observed for Vero cells, as suggested by the band intensity on agarose gel, indicating lower levels of transcription of these genes in the human leukocytes.

### Transendothelial Migration of Leukocytes Infected With NiV

**FIGURE 3:**
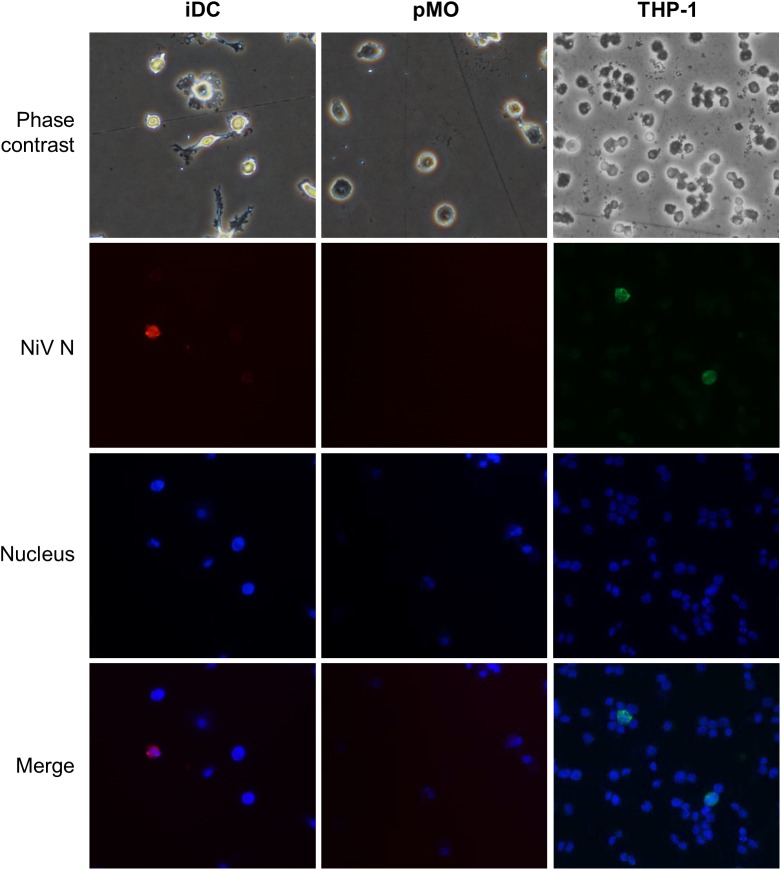
Representative images of phase contrast and immunofluorescence analyses of iDC, pMO and THP-1 infected with NiV at 48 h p.i. The infected cells were stained using polyclonal anti-NiV N with Alexa Fluor^®^ 564-conjugated secondary antibody (red) for iDC and pMO, and Alexa Fluor^®^ 488-conjugated secondary antibody (green) for THP-1. The use of the respective dyes produced less background. Cell nuclei were counterstained with Hoechst 33324 (blue). Magnification at 200×.

The transendothelial migration activity of mock-infected leukocytes and leukocytes infected with NiV were evaluated using an *in vitro* BBB model. The leukocytes were either mock-infected or infected with NiV for 48 h prior to their use in the transendothelial migration assay. At 48 h p.i., the NiV-infected leukocytes were examined for the presence of NiV N. Only NiV-infected iDC and THP-1 were positively-stained with antibody against the viral N protein (Figure 3). The number of NiV-infected leukocytes was estimated to be less than 2% as viewed from more than three high powered fields (200× magnification). Visual examination of phase contrast images showed that there were no apparent CPE observed in the positively-stained leukocytes which were infected with NiV. No positively-stained cells were observed for pMO infected with NiV.

**FIGURE 4:**
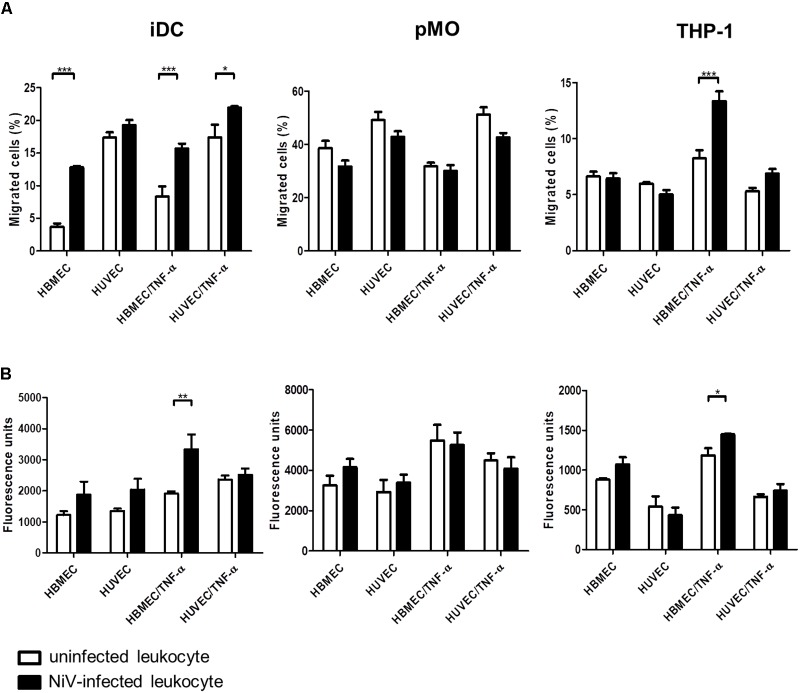
Transendothelial migration of iDC, pMO, and THP-1, either mock-infected or infected with NiV, across untreated and TNF-α-treated HBMEC and HUVEC: **(A)** migration was presented as the percentage of migrated cells [represented on the y-axis as migrated cells (%)], and **(B)** permeability of endothelial monolayers after transendothelial migration as measured by the flux of 70 kDa fluorescein-dextran (represented on the y-axis as fluorescence units). Values are the average of three biological replicates and error bars indicate standard deviation (^∗^*p* < 0.05, ^∗∗^*p* < 0.01, ^∗∗∗^*p* < 0.001). Data is representative of two independent biological replicates.

The *in vitro* BBB model was established using HBMEC. For comparisons, endothelial cells of a different origin, HUVEC, was used to establish the *in vitro* BBB model in place of HBMEC. The average TEER values achieved for the *in vitro* BBB models were 91.5 ± 16.2 Ωcm^2^ for HBMEC and 70.8 ± 10.1 Ωcm^2^ for HUVEC, with the highest TEER values being 125.1 and 93.7 Ωcm^2^, respectively. The transendothelial migration results of the NiV-infected leukocytes are presented in Figure 4A. Overall, pMO had the highest migration rate of 30–50% across either HBMEC or HUVEC, while iDC and THP-1 migrated at around 5–20%. HBMEC was more restrictive than HUVEC to the migration of iDC and pMO, either mock-infected or infected with NiV. Approximately three times more NiV-infected iDC than mock-infected iDC migrated across HBMEC (*p* < 0.001), although no significant increase in migration was observed across HUVEC. NiV infection of THP-1 had similar migration rates to mock-infected THP-1 across both HBMEC and HUVEC. This was, similarly, observed for migration of mock-infected pMO and pMO infected with NiV across HBMEC and HUVEC.

With TNF-α treatment of the endothelial cell monolayer to mimic an inflammatory response in NiV infection (Rockx et al., 2011; Munster et al., 2012; Gupta et al., 2013), migration rates increased for iDC and THP-1 (Figure 4A). A significant increase in migration of NiV-infected iDC was noted across HBMEC/TNF-α and HUVEC/TNF-α (*p* < 0.001 and *p* < 0.05, respectively) when compared to mock-infected iDC. Similarly, migration of NiV-infected THP-1 almost doubled across HBMEC/TNF-α (*p* < 0.001) and increased slightly across HUVEC/TNF-α (not significant) when compared to mock-infected THP-1. In contrast, migration rates for pMO infected with NiV remained similar to that of mock-infected pMO across HBMEC/TNF-α and HUVEC/TNF-α.

### Permeability of Endothelial Cells Post-migration

The permeability of endothelial cell monolayer of the *in vitro* BBB model was determined immediately after transendothelial migration assay (Figure 4B) as evaluated based on the amount of fluorescein-dextran that passed through the endothelial cell monolayer in the transwell after migration. The permeability of HBMEC and HUVEC increased post-migration with NiV-infected iDC compared to mock-infected iDC. Across TNF-α-treated endothelial cells, permeability of HBMEC/TNF-α was significantly increased post-migration with NiV-infected iDC (*p* < 0.01), but remained unchanged for HUVEC/TNF-α, when compared to post-migration with mock-infected iDC. In post-migration with NiV-infected THP-1, there was an increase in the permeability of HBMEC and HBMEC/TNF-α, although only the latter was significant (*p* < 0.05) when compared to mock-infected THP-1. For pMO, which did not support active NiV replication, there were no significant changes in permeability of both HBMEC and HUVEC post-migration with mock-infected and pMO infected with NiV.

### Overlay on Vero Cell Monolayer

**FIGURE 5:**
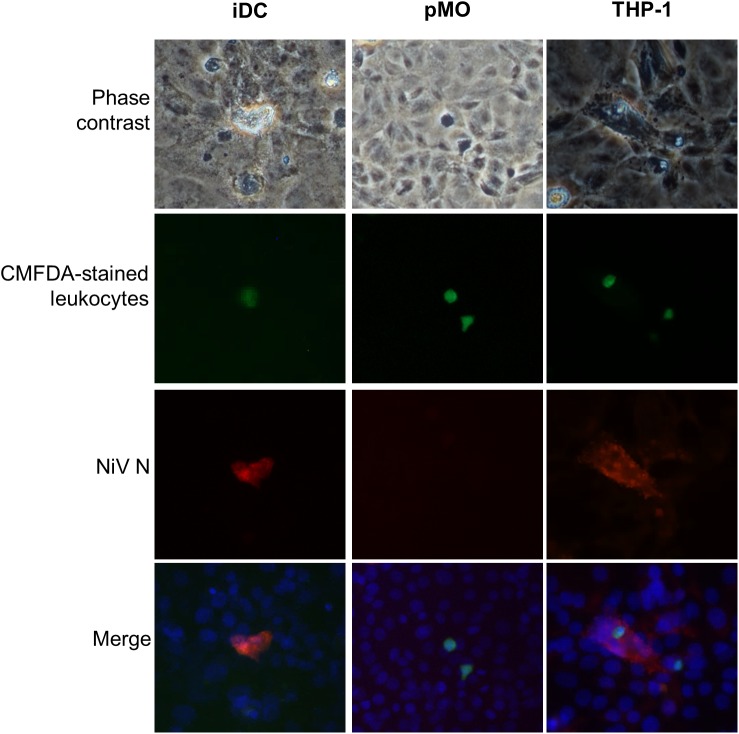
Representative images of migrated iDC, pMO, and THP-1 overlaid on Vero cell monolayer. The CMFDA dye-stained migrated NiV-infected leukocytes (green) were overlaid on Vero cells and immunostained after 24 h by incubating with polyclonal anti-NiV N and Alexa Fluor^®^ 564-conjugated secondary antibody (red). Cell nuclei were counterstained with Hoechst 33324 (blue). Magnification at 200×.

The migrated leukocytes infected with NiV were evaluated if they could infect other susceptible cells. The migrated leukocytes collected from the lower chamber of the transwell of the *in vitro* BBB model were overlaid onto a monolayer of Vero cells which were grown in Falcon culture slides (Figure 5). With migrated NiV-infected iDC and THP-1, both the leukocytes and the Vero cells beneath it were positively stained for NiV N, however, this was rarely observed due to the low percentage of NiV-infected iDC and THP-1. In contrast, with migrated pMO infected with NiV, there was no positive staining for NiV N in pMO or the Vero cells.

## Discussion

The hematogenous route has been suggested for the multi-organ spread of NiV. This has been evidenced by low level viremia and widespread systemic vasculitis in NiV patients (Wong et al., 2002). Studies on NiV infection in animal models have also demonstrated low level viremia. In infected pigs, cats and African green monkeys, cell-free viremia contributed to NiV spread (Middleton et al., 2002; Weingartl et al., 2005; Geisbert et al., 2010). On the other hand, NiV spread by cell-associated viremia was demonstrated in NiV-infected pigs (Stachowiak and Weingartl, 2012), and in hamsters and *in vitro* using human leukocytes (Mathieu et al., 2011), with findings that leukocytes were productively infected which contributed to viremia. Alternatively, leukocytes could act as a mechanical transporter of NiV as lymphocytes were found to bind NiV on their surface, without themselves being infected, and transferred NiV to other cells upon contact (Mathieu et al., 2011). These surface-bound NiV were not internalized and could stay bound on the lymphocyte cell surface for as long as 4 days without much loss of infectivity. This lymphocyte-mediated *trans*-infection was demonstrated *in vitro* and *in vivo* in hamsters (Mathieu et al., 2011). Besides NiV, other paramyxoviruses including Tioman virus (Yaiw et al., 2008), measles virus (Ludlow et al., 2009; Singh et al., 2016), and canine distemper virus (Rudd et al., 2006) have been reported to infect leukocytes and may utilize them for systemic spread of the virus.

This present study examined the role of human leukocytes in the dissemination of NiV. NiV infection was previously established in human monocytic cell line THP-1 (Chang et al., 2006), and this cell type was hypothesized to transport NiV across the BBB into the CNS. Here, we found primary iDC and THP-1, but not pMO, were permissive to NiV and supported low level NiV replication. At 0 h p.i., high intracellular viral titers were observed in NiV-infected iDC and NiV-infected THP-1, but not pMO infected with NiV, suggesting that iDC and THP-1 had high affinity for NiV. Mathieu et al. (2011) previously reported human leukocytes could efficiently bind NiV, with dendritic cells having the second highest surface binding of NiV after lymphocytes. In this study, the high binding, however, did not correlate with the resulting NiV replication kinetics in the cells. NiV was not able to replicate efficiently in iDC and THP-1 as observed from the continual but low increase in extracellular viral titers. Intracellular viral titers in these cells also reflected poor replication levels of NiV. Of note was the large decrease in intracellular titer in iDC observed between 0 and 24 h p.i., which was not observed in THP-1. This decrease suggested that a large number of NiV was bound successfully on iDC but could not replicate efficiently once internalized, possibly due to the virus being processed for antigen presentation since dendritic cells are phagocytes (Savina and Amigorena, 2007), and therefore leaving only a small number of infectious NiV to initiate productive infection in iDC (Stachowiak and Weingartl, 2012). This may also explain the poor levels of intracellular viral titer in NiV-infected THP-1, as THP-1 is phagocytic in nature. The low levels of viral replication observed here is similar to that previously reported in dendritic cells and THP-1 (Chang et al., 2006; Mathieu et al., 2011; Gupta et al., 2013). It was interesting to note that while THP-1, a monocytic cell line, could support NiV replication, pMO could not.

The mRNA expression for the primary NiV entry receptor ephrinB2 and the alternative receptor ephrinB3 were examined. EphrinB2 mRNA was detected in all three leukocytes used here, but ephrinB3 was noted in iDC and THP-1 only. Mathieu et al. (2011) reported mRNA expression for ephrinB2 but undetectable ephrinB3 in human dendritic cells and monocytes. In contrast, Gupta et al. (2013) reported ephrinB3 surface protein expression on dendritic cells but not ephrinB2. Here, both pMO and THP-1 could be susceptible to NiV but only the latter was permissive to NiV, suggesting that expression of viral entry receptors do not necessarily translate to permissiveness. Porcine B lymphocytes, even when stimulated to express ephrinB2, remained non-permissive to NiV, while monocytes and natural killer lymphocytes could be infected in their resting state where ephrinB2 was not expressed (Stachowiak and Weingartl, 2012). Mathieu et al. (2011) reported the non-permissiveness of primary monocytes to NiV despite detection of ephrinB2 expression in the cells. In the same report, only dendritic cells among the human leukocytes examined were found to be permissive to NiV. Taken together, it is possible that NiV infection of leukocytes is species-specific. The human leukocytes used in this study were NiV-permissive, specifically iDC and THP-1, but the virus does not appear to be replicating efficiently in these cells.

This present study also describes for the first time the role of NiV-infected leukocytes in viral spread by looking at changes in transendothelial migration activity. The TEER values of the *in vitro* BBB model established in this study were comparable to other studies using HBMEC (Jong et al., 2001; Man et al., 2008; Paradis et al., 2016) and HUVEC (Wang et al., 2004; Man et al., 2008) in monoculture models in transwells. Values ranging from 150 to 200 Ωcm^2^ for HBMEC has been suggested for studies on permeability or transport of molecules in the BBB (Deli, 2007; Wilhelm et al., 2011), although requirements for barrier tightness in BBB leukocyte transmigration assay is not clear (Takeshita and Ransohoff, 2012). The chemoattractants RANTES and MCP-1 used in this study have been reported as part of the chemokines produced in infectious CNS pathology (Glabinski and Ransohoff, 1999), and has been found to be expressed by NiV-infected endothelial and epithelial cells (Lo et al., 2010; Lo et al., 2012; Escaffre et al., 2016).

The transendothelial migration assay demonstrated that HBMEC was more restrictive than HUVEC to migration, regardless of whether the leukocytes were infected with NiV. Restriction to migration across HBMEC was expected due to the tighter monolayer formed by HBMEC, and this was reflected in the migration rates of iDC and pMO. THP-1 showed similar migration rates across both endothelial cell types. This similar rate of migration is unlikely due to a “leaky” barrier during the set-up of the *in vitro* BBB model as TEER readings were consistent throughout. As THP-1 is a continuous cell line, it may respond differently than the primary cells used (iDC and pMO). In HBMEC, NiV-infected leukocytes that supported active viral replication, specifically iDC, had significantly higher migration rates. In contrast, there was no difference in migration rates observed between mock-infected and pMO infected with NiV. Therefore, we suggest that active NiV replication may be required to alter the transendothelial migration activity. This could be due to changes in the expression of proteins that regulate the migration process (Eugenin et al., 2006; Dittmar et al., 2008). Migration across TNF-α-treated endothelial cells was also examined as TNF-α expression is associated with CNS neuroinflammation (Glabinski and Ransohoff, 1999), and increase in TNF-α production was reported in NiV infection *in vitro* and *in vivo* (Rockx et al., 2011; Munster et al., 2012; Gupta et al., 2013). Here, TNF-α-treated endothelial cells allowed greater migration of NiV-infected leukocytes than across untreated endothelial cells, which was noted for iDC and THP-1. Migration of NiV-infected leukocytes, notably iDC, was also dependent on endothelial cell type where higher migration occurred across HBMEC/TNF-α than HUVEC/TNF-α. Migration of mock-infected and pMO infected with NiV across untreated and TNF-α-treated endothelial cells were similar, and this was likely due to the non-permissiveness of pMO to NiV infection.

Migration of NiV-infected iDC and THP-1 resulted in an increase in permeability of both endothelial cell types, particularly across HBMEC and HBMEC/TNF-α. It is possible that migration of NiV-infected leukocytes may eventually contribute to endothelial barrier disruption. This was not observed post-migration with mock-infected and pMO infected with NiV. The increase in HBMEC and HUVEC permeability post-migration caused by direct cell-free NiV infection of the endothelial cells was ruled out as the NiV-infected leukocytes were washed prior to being used for migration assay. Moreover, plaque assay was performed using the final wash supernatant and no free NiV was present (data not shown). On the other hand, increase in permeability due to NiV infection of the endothelial cell monolayer by the virus transferred from infected leukocytes was also unlikely as differences in permeability of the cell monolayers after migration of mock-infected or NiV-infected leukocytes were primarily not significant. Besides, Erbar and Maisner (2010) reported that NiV infection of porcine brain microvascular endothelial cells did not significantly affect the endothelial barrier properties early in infection at 6 h p.i., the same period in which migration was allowed to occur in this study. Additionally, in porcine (Weingartl et al., 2005) and hamster (Munster et al., 2012) models, vasculitis in the CNS was rarely seen and was observed only in late stage of infection in the hamster model. This supports our hypothesis that NiV spread to the CNS during early infection could be via infiltration of infected leukocytes across the BBB in the absence of BBB disruption. Once migrated, NiV-infected leukocytes are presumed to go on to infect surrounding tissues, either through the release of new infectious virions or by cell-to-cell contact.

The immunofluorescence analysis of migrated NiV-infected iDC and THP-1 overlaid on Vero cells showed that the migrated leukocytes could infect susceptible cells. This observation was quite rare and a method of enriching the migrated infected cells would help to better visualize the possibility of cell-to-cell spread of the virus, however, it could not be performed due to the limitations of infrastructure. Nevertheless, the lack of a widespread infection of the Vero cells suggests that infection was not due to release of infectious NiV, but rather through cell-to-cell contact. This cell-to-cell mode of infection has also been observed for the transfer of measles virus from infected monocyte-derived macrophage and infected dendritic cells to epithelial cells (Singh et al., 2016). Cell-to-cell contact, but not syncytial formation and extracellular virus release, was found to be important for measles virus infection of neuronal cells (Lawrence et al., 2000). Additionally, magnetic resonance images of NiV patients in the acute stage of the disease showed focal lesions throughout the brain, and human autopsy samples showed that infected neurons were closely associated with necrotic plaques and vasculitic vessels (Goh et al., 2000; Lim et al., 2000; Wong et al., 2002). These suggest that NiV could spread via cell-to-cell after breaching the BBB. Once in the CNS, cell-to-cell spread of NiV from infected leukocytes to surrounding cells could contribute to the focused lesions with an absence or minimum virus release. The preference of migrating across activated HBMEC over HUVEC may also add support to the observed CNS pathology in humans infected with NiV (Wong et al., 2002). Further experiments with endothelial cell of different origins could help identify if there is a preference for brain endothelial cells.

The results presented here support the role of leukocytes, specifically iDC, in the dissemination of NiV across the BBB, where viral infection enhanced transendothelial migration of the leukocytes, allowing NiV from infected leukocytes to cause infection the CNS via the “Trojan horse” mechanism. Although only a small percentage of leukocytes were infected (<2%), the percentage of migrated NiV-infected leukocytes was higher than that. This suggests that there may be other released factors from NiV-infected leukocytes that could influence their migration activity or the endothelial permeability, which should be further investigated, as well as any cellular changes caused by NiV infection in the leukocytes. The *in vitro* BBB model successfully established in this study was a monoculture. Co-culture models established with more than one cell type to accurately reflect the complex nature of actual BBB can be explored in future migration studies to better determine the leukocyte–BBB interactions. Infection of leukocytes with inactivated NiV would also help confirm if active viral replication is required to alter migration behavior, however, this was not feasible due to limited resources in the BSL3 facility. Finally, our results demonstrated that *in vitro* NiV infection of leukocytes can result in a change in cellular behavior, in this case, a change in transendothelial migration activity. Adapting these findings to animal infection models to demonstrate NiV dissemination by infected leukocytes would further support our hypothesis. In conclusion, the findings presented here implicates the ability of NiV to utilize circulating immune cells for its dissemination to the CNS and elsewhere in the body, and contributes to a better understanding of NiV pathophysiology.

## Ethics Statement

This study was carried out in accordance with the recommendations of the Medical Ethics Committee of University Malaya Medical Center with written informed consent from all subjects. All subjects gave written informed consent in accordance with the Declaration of Helsinki. The protocol was approved by the Medical Ethics Committee of the University Malaya Medical Center (MECID No. 20164-2360).

## Author Contributions

L-YC, SA, and VT conceived and designed the study. VT and M-HS performed the experiments. VT, L-YC, WW, and SA analyzed the data and revised the manuscript. VT wrote the first draft of the manuscript. All authors contributed to manuscript revision, read and approved the submitted version.

## Conflict of Interest Statement

The authors declare that the research was conducted in the absence of any commercial or financial relationships that could be construed as a potential conflict of interest.
