# Supplementary material for: Nipah Virus Infection of Immature Dendritic Cells Increases Its Transendothelial Migration Across Human Brain Microvascular Endothelial Cells
Source: Front Microbiol. 2018 Nov 13;9:2747. doi: 10.3389/fmicb.2018.02747 (PMC6244409; doi:10.3389/fmicb.2018.02747)

Band intensity analysis of ephrinB2 and B3 band intensities in Figure 2 using ImageJ software (National Institutes of Health, USA)

A. Band intensity analysis of respective amplified bands:

	Vero	iDC	pMO	THP-1	Background
ephrinB2	29530.91	1593.255	8425.238	13332.14	
ephrinB3	25900.39	9612.38	345.799	17968.34	
B-actin	42204.69	29126.83	19754.32	24856.45	226.263

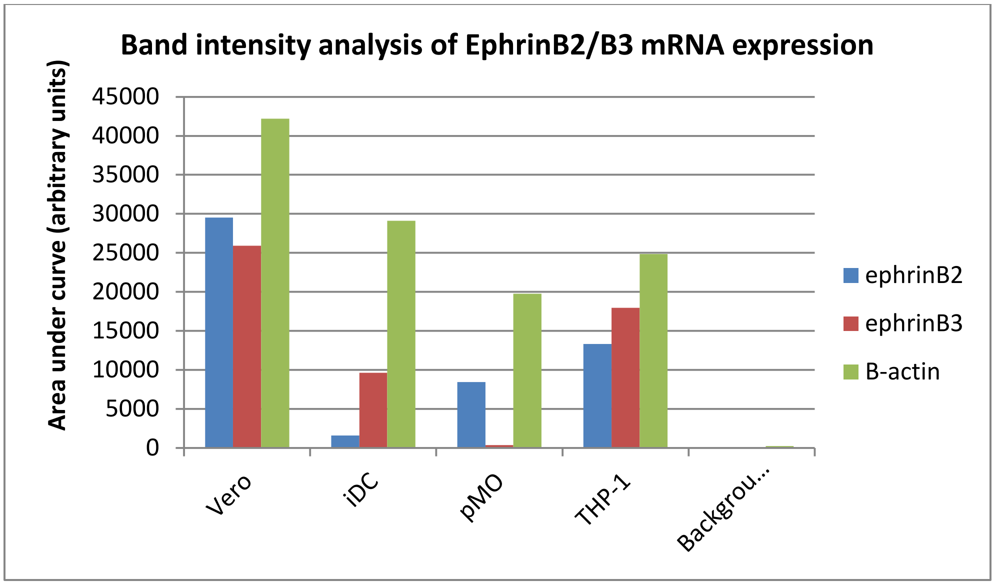

B. Band intensity of ephrinB2/B3 normalized to respective β-actin mRNA expression:

	Vero	iDC	pMO	THP-1
ephrinB2	0.700	0.055	0.427	0.536
ephrinB3	0.614	0.330	0.018	0.723

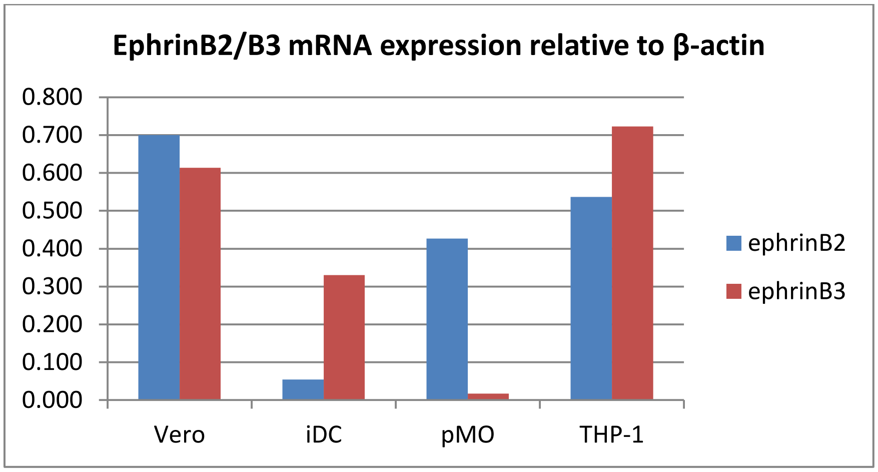

Supplement: Supplementary file 1 [file Data_Sheet_1.PDF]
