# Supplementary figures and images for: Nipah Virus Infection of Immature Dendritic Cells Increases Its Transendothelial Migration Across Human Brain Microvascular Endothelial Cells
Source: Front Microbiol. 2018 Nov 13;9:2747. doi: 10.3389/fmicb.2018.02747 (PMC6244409; doi:10.3389/fmicb.2018.02747)

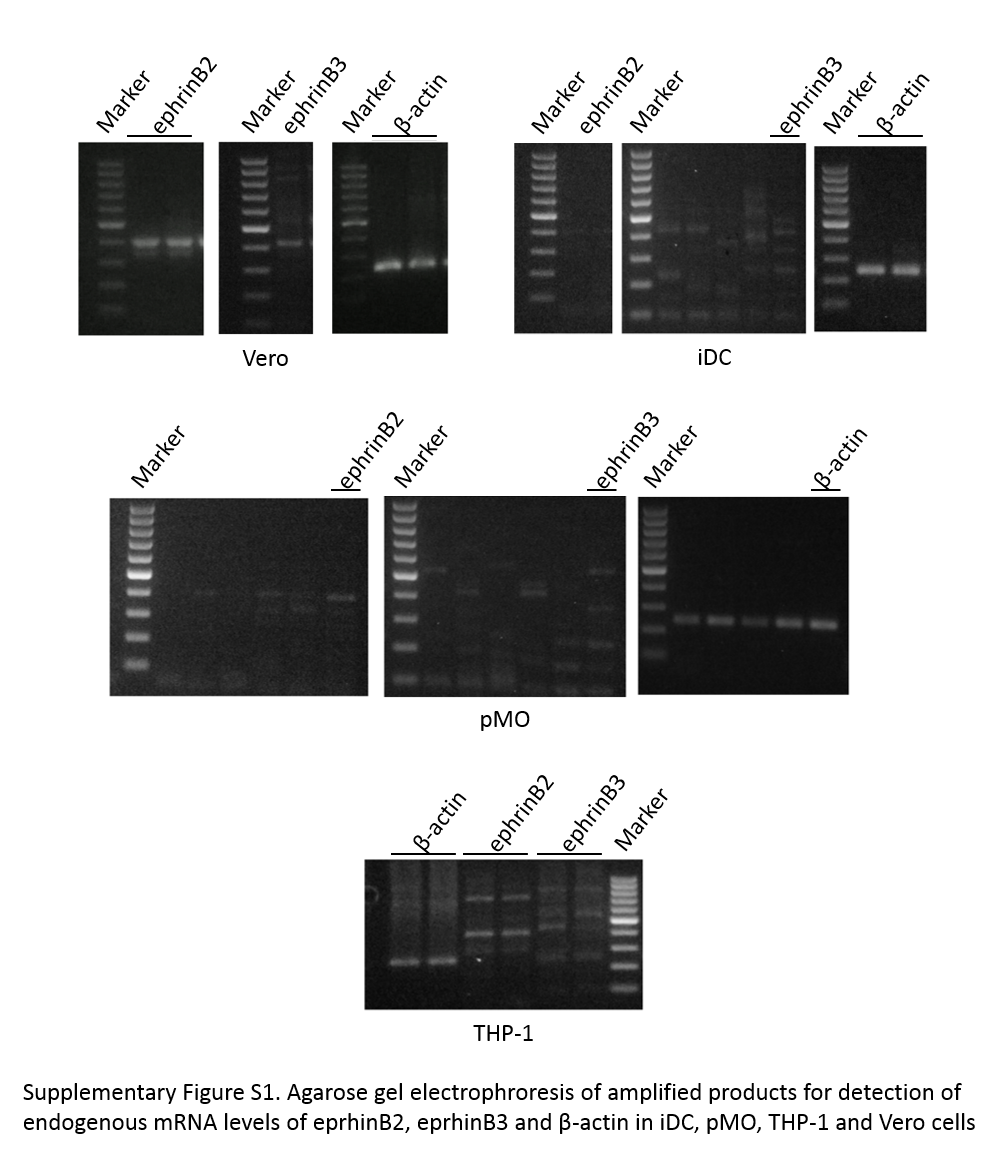

Supplement: Supplementary file 2 [file Image_1.tif]
